# Atomistic simulation of olefin polymerization reaction by organometallic catalyst: significant role of microscopic structural dynamics of (pyridylamido) Hf(IV) complex in catalytic reactivity

**DOI:** 10.3389/fchem.2025.1618025

**Published:** 2025-06-10

**Authors:** Kentaro Matsumoto, Nana Misawa, Shuhei Kanesato, Masataka Nagaoka

**Affiliations:** ^1^ Graduate School of Informatics, Nagoya University, Nagoya, Japan; ^2^ Institute of Innovation for Future Society, Nagoya University, Nagoya, Japan; ^3^ Core Research for Evolutional Science and Technology, Japan Science and Technology Agency (JST-CREST), Kawaguchi, Japan; ^4^ Element Strategy Initiative for Catalysts and Batteries (ESICB), Kyoto University, Kyoto, Japan

**Keywords:** polymerization, ion pair, polyolefin, molecular dynamics, red moon method, (pyridylamido) Hf(IV)

## Abstract

Understanding the microscopic catalytic mechanism of the olefin polymerization reaction is crucial for the rational design of next-generation catalysts. However, the dynamic nature of the active species, including the fluctuations of the ion pair structure and the orientation of substituents, presents significant challenges for theoretical approaches. In this paper, we present an overview of our recent computational studies on the role of the structural dynamics of the active species of olefin polymerization catalyst in determining reactivity, especially focusing on a novel olefin polymerization catalyst (pyridylamido) Hf(IV) complex. Utilizing the molecular dynamics method and our Red Moon method, a novel methodology we have developed for atomistic simulation of complex chemical reaction systems, we elucidate how the dynamic features, including anion coordination and steric interaction, govern the reactivity in key steps such as ligand modification and propagation reactions. In addition, we demonstrate how machine learning techniques can be applied to extract chemically meaningful descriptors from the structural ensemble obtained from atomistic simulation data of complex chemical reaction systems, thereby identifying the substituents that play an important role in propagation reactions. Our studies highlight the importance of incorporating molecular-level dynamic features of catalysts into mechanistic models.

## 1 Introduction

In today’s society, polyolefins are the most extensively used polymer resins. Consequently, significant efforts have been made to develop the olefin polymerization catalyst for achieving efficient production and precise control over the microscopic structures of resulting polymers (Sinn et al., 1980; Gibson and Spitzmesser, 2003; Wilke, 2003; Baier et al., 2014). Although gaining a precise understanding of the polymerization mechanism is crucial for catalyst development, experimentally capturing the microscopic processes remains challenging due to the complexity of the reaction and the difficulty of isolating intermediates. Therefore, computational approaches have been widely adopted to investigate the detailed reaction mechanism that cannot be easily probed experimentally. For example, the reaction mechanisms have been extensively investigated using quantum chemical methods from a static point of view, particularly with respect to the origin of monomer reactivity, as well as regio- and stereoselectivity (Kawamura-Kuribayashi et al., 1992; Alt and Köppl, 2000; Angermund et al., 2000; Lanza et al., 2000; Lanza et al., 2001; Rappé et al., 2000; Resconi et al., 2000; Zurek and Ziegler, 2003; Ziegler et al., 2005; Motta et al., 2007; Motta et al., 2008; Tomasi et al., 2007; De Rosa et al., 2016). While these quantum mechanical methods provide valuable insights into the static features of the reaction mechanism, understanding the dynamic aspect of the catalytic processes requires a different set of computational tools. In this regard, various methodologies have been developed. For instance, *ab initio* molecular dynamics and molecular dynamics using machine learning potentials (MLPs), the latter of which has significantly progressed in recent years (Unke et al., 2021), have been widely used. However, bridging the gap between the timescales of chemical reactions and molecular motions remains challenging due to the high computational cost; the former, which involves the bond formation and breaking, occurs far less frequently than the latter. Another important class of approaches is reactive force fields, such as ReaxFF (van Duin et al., 2001; Senftle et al., 2016), which have been successfully applied to a wide range of systems. However, reactive force fields are sometimes difficult to parametrize accurately (Gissinger et al., 2017) and suffer from limited transferability of their parameters (Senftle et al., 2016). For these reasons, studies addressing the dynamic aspect of the polymerization reaction with organometallic catalyst remain relatively scarce, even though such dynamics are crucial for a comprehensive understanding of the catalytic behavior (Correa and Cavallo, 2006; Yang and Ziegler, 2006; Rowley and Woo, 2011).

Under such circumstances, we have investigated the microscopic dynamics of the active species and its role in the olefin polymerization reaction by employing the molecular dynamics (MD) method and the Red Moon (RM) method, a novel methodology we have developed for atomistic simulation of complex chemical reaction systems. In particular, we have focused on the (pyridylamido) Hf(IV) complex, which attracts much attention as a novel catalyst with high activity (Boussie et al., 2003; Boussie et al., 2006; Chum and Swogger, 2008; Frazier et al., 2011). In this mini-review, we present an overview of our recent computational investigations, highlighting the dynamic features of the active species and their influence on the olefin polymerization reaction (Matsumoto et al., 2016; Matsumoto et al., 2019; Misawa et al., 2021; Misawa et al., 2023; Kanesato et al., 2023; Kanesato et al., 2024).

**FIGURE 1:**
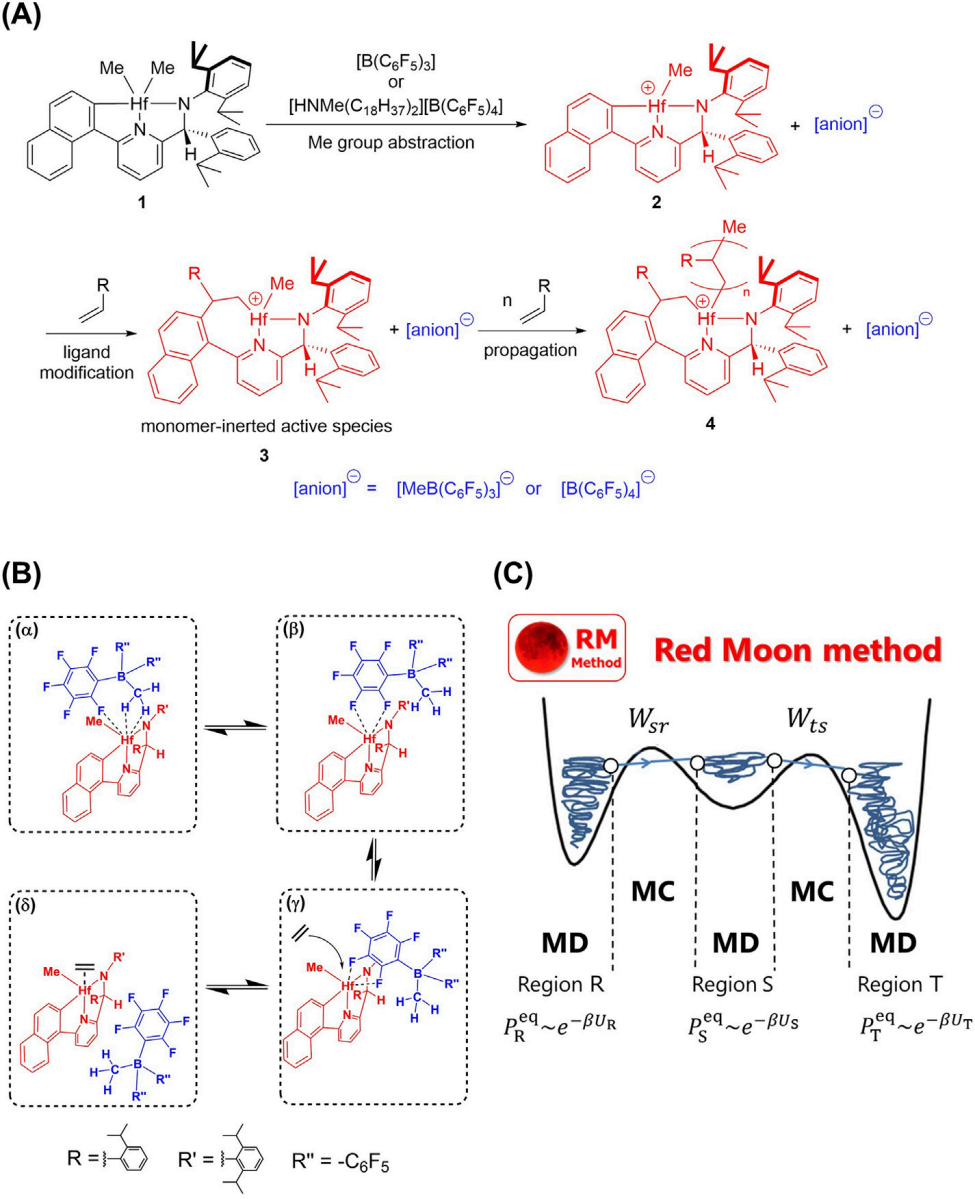
**(A)** Schematic representation of activation (Me group abstraction and ligand modification) and propagation reaction of (pyridylamido) Hf(IV) complex. **(B)** Schematic representation of the associative active site opening mechanism. Adapted with permission from Organometallics, 2016, 35, 24, 4099–4105. Copyright 2016 American Chemical Society. **(C)** A schematic representation of the Red Moon method. In three regions R, S,and T, the configurational distribution $P_{x}^{\text{eq}}\left(x=R,S,T\right)$ is proportional to the exponential factor $e^{-\beta U_{x}}$ , where $U_{x}$ is the potential function in each region. $W_{sr}$ and $W_{ts}$ are the transition probabilities from a configuration state r in region R to s in region S, and from s in region S to t in region T, respectively.

Similar to other olefin polymerization catalysts with group 4 metal, (pyridylamido) Hf(IV) complex **1** requires an activation process. As shown in Figure 1A, when neutral complex **1** reacts with such as [B(C_6_F_5_)_3_] or [HNMe(C_18_H_37_)_2_][B(C_6_F_5_)_4_], called cocatalyst, one of the Me groups is abstracted and results in the ion pair (IP) of the cation **2** and the anion. Afterwards, monomeric olefin is inserted into the Hf-C_aryl_ bond, which is known as ligand modification reaction. It is widely accepted that the **3** referred to as “monomer-inserted active species” is the genuine active species for the following propagation reaction (Froese et al., 2007; Zuccaccia et al., 2009).

This mini-review is organized as follows. In Section 2, we present molecular dynamics study on the structural dynamics of the active species in the ligand modification reaction. We revealed a characteristic dynamic of the anion dissociation from the active site, which we refer to as the associative active site opening (AASO) mechanism. Next, in Section 3, we focus on the structural dynamics of the active species in the propagation reaction. For this purpose, we utilized the RM method. Our study reasonably reproduced the anion-dependent reactivity of the catalyst and clearly illustrated how the structural dynamics of the active species is interwoven with the propagation reaction. Then, in Section 4, we present an application of our RM method, especially focusing on the role of structural dynamics in the mechanism of steric hindrance. By combining machine learning techniques, we successfully identified the key substituents and elucidated how they govern the steric hindrance around the active site. Finally, in Section 5, we conclude with emphasis on the importance of capturing dynamic features of the active species in mechanistic analysis of olefin polymerization reaction.

## 2 Associative active site opening mechanism in the ligand modification reaction

Experimental evidence indicates that the active site, i.e., the Hf atom on the cation species **2** is occupied by the anion due to its strong interaction with the Hf atom (Zuccaccia et al., 2008; Zuccaccia et al., 2009). Thus, the anion dissociation from the active site is a prerequisite for the ligand modification reaction. However, active site opening process is hard to statically investigate with such as quantum mechanical method because it is a dynamic process. We, therefore, developed a molecular model of the IP of the cation **2** and the counter anion [MeB(C_6_F_5_)_3_]^−^, and investigated its structural dynamics by MD method (Matsumoto et al., 2016).

From our simulation, it was revealed that the counter anion exhibits characteristic dissociation from the active site when monomeric ethylene molecules are present in the system (Figure 1B). Initially, the counter anion [MeB(C_6_F_5_)_3_]^−^ interacts with the Hf atom using a single F atom and Me group (structure α in Figure 1B). Subsequently, the anion interacts with the Hf atom solely via F atoms, leading to the structure β in Figure 1B. Then, the borate anion can move along the cation’s surface while staying coordinated to the Hf atom, thereby yielding enough coordination space for the monomeric ethylene to access the active site (structure γ in Figure 1B). Such a behavior arises from the planar geometry, and the orthogonal alignment of pyridylamide ligand and perfluorophenyl group. In the final step, the anion dissociation and the monomer coordination occur associatively (structure δ in Figure 1B), which we refer to as the associative active site opening (AASO).

According to the free energy barrier, structural change from structure α to β in Figure 1B is the slowest step in the AASO mechanism, involving the Me group dissociation. It is inferred, therefore, that the ligand modification occurs more rapidly if the anion is [B(C_6_F_5_)_4_]^−^ because of the lack of Me group strongly interacting with the active site. In fact, it is experimentally shown that the polymer growth initiation proceeds more slowly with [MeB(C_6_F_5_)_3_]^−^ (Cueny et al., 2017), which supports the validity of the AASO mechanism.

## 3 Structural dynamics of the ion pair active species interwoven with the propagation reaction

The anion-dependent reactivity is also observed in the propagation as well as in the ligand-modification. In fact, it has been experimentally observed that active species **3** with [B(C_6_F_5_)_4_]^−^ tends to show higher polymerization rate of 1-octene than that with [MeB(C_6_F_5_)_3_]^−^ (Cueny et al., 2017), which indicates that the dynamic features of the IP active species has an effect on the propagation reaction as in the case of the ligand modification. However, it is still challenging to computationally investigate how the propagation reaction and the IP dynamics influence each other because these phenomena differ significantly in timescale. The former, characterized by the formation and breaking of chemical bonds, takes place much less frequently than the structural changes seen in the latter. To tackle this problem, the RM method (Nagaoka et al., 2013; Nagaoka et al., 2019; Nagaoka, 2024) was employed. In the RM method, the molecular motions over a relatively short time scale are handled with the MD method, while the chemical reaction processes involving formation and breaking of chemical bonds over a relatively long time scale are handled with Monte Carlo (MC) method. A single cycle comprising these two methods is referred to as the “RM cycle”. Repeating the RM cycle allows stochastic simulation of a series of propagations (Figure 1C). Moreover, by employing the time transformation theory (Suzuki and Nagaoka, 2017; Nagaoka et al., 2019), the RM cycle is mapped onto an effective real-time domain. Some independent groups have recently adopted the same spirit of the RM method and reasonably applied their methods in the field of lithium-ion batteries (Biedermann et al., 2021b; Biedermann et al., 2021a; Abbott and Hanke, 2022). Furthermore, Okabe et al. employed a treatment similar to the RM method to primarily study the cross-linking reactions and physical properties of epoxy resins (Takaba et al., 2008; Okabe et al., 2013; Oya et al., 2021). We believe that these works further support the validity of the direction pursued by our RM method.

**FIGURE 2:**
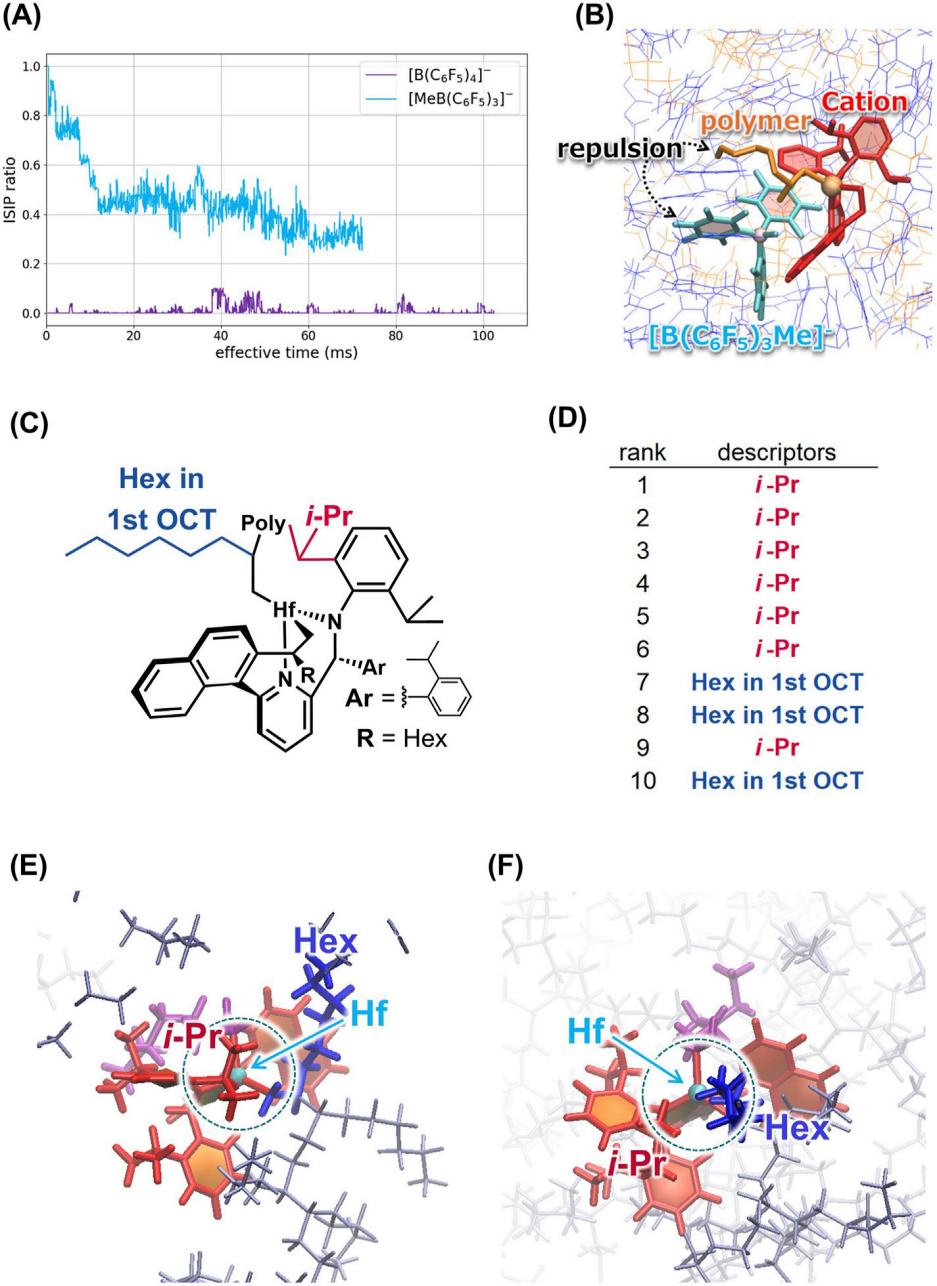
**(A)** Averaged ISIP ratio as a function of the effective real-time in each IP system. **(B)** OSIP structure of the IP system with [MeB(C_6_F_5_)_3_]^−^ anion after the first monomer insertion, where the steric hindrance between the inserted monomer and the counter anion induces the structural transition from the ISIP to OSIP. Adapted with permission from J. Phys. Chem. B 2023, 127, 5, 1209–1218. Copyright 2023 American Chemical Society. **(C)** Schematic representation of the cation showing the *i*-Pr and Hex groups in the 1-octene unit adjacent to the Hf atom (Hex in 1st OCT) which affect the propagation reaction. **(D)** Results of descriptor extraction with machine learning techniques. Typical snapshots of steric hindrance around the active site due to **(E)** the *i*-Pr group and **(F)** the Hex group. Adapted with permission from Phys. Chem. B 2024, 128, 25, 6178–6188. Copyright 2024 American Chemical Society.

By applying the RM method, higher 1-octene consumption with [B(C_6_F_5_)_4_]^−^ was reasonably reproduced (Misawa et al., 2021; Misawa et al., 2023). Furthermore, to reveal the dynamic features of the IPs, according to the location of the counter anion relative to the cation, we classified the IP structure into two classes, that is, inner-sphere IP (ISIP) state where the counter anion is coordinated to the Hf atom, and the outer-sphere IP (OSIP) where the counter anion is dissociated from the Hf atom. Figure 2A illustrates the variations of the ISIP ratio averaged over the 10 trajectories obtained from the RM simulation. It is clearly shown that the IP of [MeB(C_6_F_5_)_3_]^−^ forms ISIP which inhibits the coordination of the monomeric 1-octene to the active site. Notably, the ISIP ratio of the IP of [MeB(C_6_F_5_)_3_]^−^ drops steeply within the first 20 ms, as the inserted monomer has the steric repulsion with the counter anion after the first monomer insertion (Figure 2B). Afterwards, the ISIP ratio fluctuates between 40% and 60%, which indicates the IP of the [MeB(C_6_F_5_)_3_]^−^ reaches a quasi-equilibrium state. By contrast, the IP of [B(C_6_F_5_)_4_]^−^ predominantly retains the OSIP across the entire simulation, leading to the faster propagation. These results reveal significant differences in the dynamic features between the two IPs and illustrate how the IP dynamics is interwoven with the propagation reaction.

## 4 Effect of the steric hindrance on the propagation reaction: extraction of essential descriptors by machine learning techniques

In general, it is well known that the steric hindrance between a catalyst’s ligands and the reacting monomers significantly affects monomer reactivity, as well as regio- and stereoselectivity in olefin polymerization reactions (Kawamura-Kuribayashi et al., 1992; Lanza et al., 2000; Lanza et al., 2001; Zurek and Ziegler, 2003; Ziegler et al., 2005; Motta et al., 2007; Motta et al., 2008; Tomasi et al., 2007; De Rosa et al., 2016). Therefore, a precise understanding of the microscopic mechanism of steric hindrance caused by substituents is essential for the rational design of catalysts that yield polymers with desired physical properties. In fact, the relationship between the catalyst structure and its reactivity has been extensively investigated using quantum mechanical methods. However, our studies presented above imply that not only the static structure of the catalyst but also its structural dynamics plays a significant role in the mechanism of steric hindrance. Motivated by these considerations, we investigated the dynamic aspect of the steric hindrance in coordinative chain transfer copolymerization of ethylene and 1-octene by (pyridylamido) Hf(IV) by combining our RM method and machine learning techniques (Kanesato et al., 2023; Kanesato et al., 2024).

To begin with, we confirmed that the frequency of the chain transfer reaction and the ethylene content in the resulting polymers obtained from our simulation are consistent with the experimental observations (Kanesato et al., 2023). These agreements validate the reliability of our simulation and the subsequent mechanistic analysis of the steric hindrance by the substituent. In addition, it is also shown that our methodology is applicable not only to homo polymerization but also to more complex polymerization systems such as coordinative chain transfer copolymerization.

Next, we attempted to find substituents that affect the propagation reaction by extracting essential descriptors using a machine learning technique. For this purpose, by using the Cartesian coordinate values of the cationic active species from our RM simulation as input variables, we developed random forest classification models to determine whether no reactant for the propagation reaction is found, or a reactant is found and the propagation reaction proceeds. We collected a large data set containing 4,146 structural entries from our RM simulation, and applied RMSD fitting to align the cationic active species, addressing the lack of rotational and translation invariance in Cartesian coordinates. The two hyperparameters, the number and depth of the trees, were optimized using grid search with 10-fold cross-validation.

Subsequently, based on the feature importance, we extracted the substituents whose Cartesian coordinate values are important. Figures 2C,D indicate that the *i*-Pr group of the cationic active species and the hexyl group of the inserted 1-octene adjacent to the Hf atom have significant effects on the occurrence of the propagation reaction. In fact, two snapshots from our RM simulation (Figures 2E,F), where no reactant for the propagation reaction is found, clearly show that the *i*-Pr group or the hexyl group occupies the active site and inhibits the approach of monomers.

It is worth noting that the combination of RM simulation and machine learning techniques successfully identified the substituents that affect the propagation reaction. This fact includes two important aspects: First, the RM simulation can provide meaningful information, including the reactions and dynamic features of the catalyst, for the complex reaction system that are hard to analyze using conventional approaches. Second, we have proposed a new scheme to analyze the chemical reaction dynamics by integrating molecular simulation and data science.

## 5 Conclusion and future perspectives

In this mini-review, we presented an overview of our recent computational studies on the role of the structural dynamics of the active species of olefin polymerization catalyst, especially focusing on the active species of (pyridylamido) Hf(IV) complex. By employing molecular dynamics method and Red Moon (RM) method, i.e., a novel methodology we have developed for the atomistic simulation of complex chemical reaction systems, we have revealed that the dynamic features such as the anion coordination and the steric interaction by the substituents significantly influence the key reaction steps, including ligand modification and propagation reactions. Furthermore, by combining machine learning techniques with our RM method, we successfully identified the substituents that govern the steric hindrance around the active site. These findings underscore the importance of capturing molecular-level dynamics in the mechanistic analysis of olefin polymerization reactions. They also demonstrate the potential of molecular simulation and a simulation-machine learning hybrid approach for uncovering the structure-reactivity relationships that are inaccessible by static models.

Although the role of structural dynamics has not been explored enough in previous mechanistic models, we find that some experimental and theoretical observations could be viewed as consistent with our perspective. For example, it has been speculated that the bulky substituents increase the propagation rate by locking the anion into a position away from the cationic metal center (Cueny et al., 2021). In addition, the rearrangement of the backbone structure of the cationic active species during the capture of a monomeric olefin has been proposed to influence the comonomer affinity (Zaccaria et al., 2017). We consider these observations to be suggestive of the underlying role of the dynamics in determining catalytic reactivity. We believe that a deeper understanding of the dynamic features of the olefin polymerization catalysts, often overlooked in static models, will become an essential component in the mechanistic understanding and contribute to the rational design of next-generation catalysts in the future.

## References

[B1] Abbott J. W. Hanke F. (2022). Kinetically corrected Monte Carlo-molecular dynamics simulations of solid electrolyte interphase growth. J. Chem. Theory Comput. 18, 925–934. 10.1021/acs.jctc.1c00921 35007421

[B2] Alt H. G. Köppl A. (2000). Effect of the nature of metallocene complexes of group IV metals on their performance in catalytic ethylene and propylene polymerization. Chem. Rev. 100, 1205–1222. 10.1021/cr9804700 11749264

[B3] Angermund K. Fink G. Jensen V. R. Kleinschmidt R. (2000). Toward quantitative prediction of stereospecificity of metallocene-based catalysts for α-olefin polymerization. Chem. Rev. 100, 1457–1470. 10.1021/cr990373m 11749271

[B4] Baier M. C. Zuideveld M. A. Mecking S. (2014). Post-metallocenes in the industrial production of polyolefins. Angew. Chem. Int. Ed. Engl. 53, 9722–9744. 10.1002/anie.201400799 25146087

[B5] Biedermann M. Diddens D. Heuer A. (2021a). Connecting the quantum and classical mechanics simulation world: applications of reactive step molecular dynamics simulations. J. Chem. Phys. 154, 194105. 10.1063/5.0048618 34240915

[B6] Biedermann M. Diddens D. Heuer A. (2021b). Rs@md: introducing reactive steps at the molecular dynamics simulation level. J. Chem. Theory Comput. 17, 1074–1085. 10.1021/acs.jctc.0c01189 33497226

[B7] Boussie T. R. Diamond G. M. Goh C. Hall K. A. LaPointe A. M. Leclerc M. (2003). A fully integrated high-throughput screening methodology for the discovery of new polyolefin catalysts: discovery of a new class of high temperature single-site group (IV) copolymerization catalysts. J. Am. Chem. Soc. 125, 4306–4317. 10.1021/ja020868k 12670253

[B8] Boussie T. R. Diamond G. M. Goh C. Hall K. A. LaPointe A. M. Leclerc M. K. (2006). Nonconventional catalysts for isotactic propene polymerization in solution developed by using high-throughput-screening technologies. Angew. Chem. Int. Ed. Engl. 45, 3278–3283. 10.1002/anie.200600240 16619317

[B9] Chum P. S. Swogger K. W. (2008). Olefin polymer technologies—history and recent progress at the dow chemical company. Prog. Polym. Sci. 33, 797–819. 10.1016/j.progpolymsci.2008.05.003

[B10] Correa A. Cavallo L. (2006). Dynamic properties of metallocenium ion pairs in solution by atomistic simulations. J. Am. Chem. Soc. 128, 10952–10959. 10.1021/ja062407v 16910692

[B11] Cueny E. S. Johnson H. C. Anding B. J. Landis C. R. (2017). Mechanistic studies of hafnium-pyridyl Amido-catalyzed 1-octene polymerization and chain transfer using quench-labeling methods. J. Am. Chem. Soc. 139, 11903–11912. 10.1021/jacs.7b05729 28763618

[B12] Cueny E. S. Nieszala M. R. Froese R. D. J. Landis C. R. (2021). Nature of the active catalyst in the hafnium-pyridyl amido-catalyzed alkene polymerization. ACS Catal. 11, 4301–4309. 10.1021/acscatal.1c00394

[B13] De Rosa C. Di Girolamo R. Talarico G. (2016). Expanding the origin of stereocontrol in propene polymerization catalysis. ACS Catal. 6, 3767–3770. 10.1021/acscatal.6b00863

[B14] Frazier K. A. Froese R. D. He Y. Klosin J. Theriault C. N. Vosejpka P. C. (2011). Pyridylamido hafnium and zirconium complexes: synthesis, dynamic behavior, and ethylene/1-octene and propylene polymerization reactions. Organometallics 30, 3318–3329. 10.1021/om200167h

[B15] Froese R. D. J. Hustad P. D. Kuhlman R. L. Wenzel T. T. (2007). Mechanism of activation of a hafnium pyridyl-amide olefin polymerization catalyst: ligand modification by monomer. J. Am. Chem. Soc. 129, 7831–7840. 10.1021/ja070718f 17542583

[B16] Gibson V. C. Spitzmesser S. K. (2003). Advances in non-metallocene olefin polymerization catalysis. Chem. Rev. 103, 283–316. 10.1021/cr980461r 12517186

[B17] Gissinger J. R. Jensen B. D. Wise K. E. (2017). Modeling chemical reactions in classical molecular dynamics simulations. Polymer 128, 211–217. 10.1016/j.polymer.2017.09.038 33149370 PMC7608055

[B18] Kanesato S. Yasoshima K. Matsumoto K. Misawa N. Suzuki Y. Koga N. (2024). Atomistic simulation of Hf-pyridyl Amido-catalyzed chain transfer alkene polymerization reaction and its machine learning for extraction of essential descriptors: effect of microscopic steric hindrance on the monomer insertion process. J. Phys. Chem. B 128, 6178–6188. 10.1021/acs.jpcb.4c01303 38845119

[B19] Kanesato S. Yasoshima K. Misawa N. Matsumoto K. Suzuki Y. Koga N. (2023). Atomistic chemical elucidation of the higher-rate reaction mechanism in Hf-pyridyl amido-catalyzed copolymerization of ethene and 1-octene: application of red moon simulation with polymer propagation diagrams. J. Phys. Chem. B 127, 7735–7747. 10.1021/acs.jpcb.3c03966 37656662 PMC10510719

[B20] Kawamura-Kuribayashi H. Koga N. Morokuma K. (1992). An *ab initio* MO and MM study of homogeneous olefin polymerization with silylene-bridged zirconocene catalyst and its regio- and stereoselectivity. J. Am. Chem. Soc. 114, 8687–8694. 10.1021/ja00048a049

[B21] Lanza G. Fragalà I. L. Marks T. J. (2000). Ligand substituent, anion, and solvation effects on ion pair structure, thermodynamic stability, and structural mobility in “constrained geometry” olefin polymerization catalysts: an *ab initio* quantum chemical investigation. J. Am. Chem. Soc. 122, 12764–12777. 10.1021/ja000571r

[B22] Lanza G. Fragalà I. L. Marks T. J. (2001). Metal and ancillary ligand structural effects on ethylene insertion processes at cationic group 4 centers. A systematic, comparative quantum chemical investigation at various *ab initio* levels. Organometallics 20, 4006–4017. 10.1021/om0102899

[B23] Matsumoto K. Sandhya K. S. Takayanagi M. Koga N. Nagaoka M. (2016). An active site opening mechanism in a (Pyridylamide)hafnium(IV) ion pair catalyst: an associative mechanism. Organometallics 35, 4099–4105. 10.1021/acs.organomet.6b00804

[B24] Matsumoto K. Takayanagi M. Suzuki Y. Koga N. Nagaoka M. (2019). Atomistic chemical computation of Olefin polymerization reaction catalyzed by (pyridylamido)hafnium(IV) complex: application of Red Moon simulation. J. Comput. Chem. 40, 421–429. 10.1002/jcc.25707 30351517

[B25] Misawa N. Matsumoto K. Suzuki Y. Saha S. Koga N. Nagaoka M. (2023). (Pyridylamido)Hf(IV)-Catalyzed 1-octene polymerization reaction interwoven with the structural dynamics of the ion-pair-active species: bridging from microscopic simulation to chemical kinetics with the red moon method. J. Phys. Chem. B 127, 1209–1218. 10.1021/acs.jpcb.2c07296 36706280

[B26] Misawa N. Suzuki Y. Matsumoto K. Saha S. Koga N. Nagaoka M. (2021). Atomistic simulation of the polymerization reaction by a (Pyridylamido)hafnium(IV) catalyst: counteranion influence on the reaction rate and the living character of the catalytic system. J. Phys. Chem. B 125, 1453–1467. 10.1021/acs.jpcb.0c10977 33502856

[B27] Motta A. Fragaà I. L. Marks T. J. (2008). Links between single-site heterogeneous and homogeneous catalysis. DFT analysis of pathways for organozirconium catalyst chemisorptive activation and olefin polymerization on gamma-alumina. J. Am. Chem. Soc. 130, 16533–16546. 10.1021/ja802439u 19554691

[B28] Motta A. Fragalà I. L. Marks T. J. (2007). Stereochemical control mechanisms in propylene polymerization mediated by C1-symmetric CGC titanium catalyst centers. J. Am. Chem. Soc. 129, 7327–7338. 10.1021/ja068990x 17511456

[B29] Nagaoka M. (2024). The computational molecular technology for complex reaction systems: the Red Moon approach. Wiley Interdiscip. Rev. Comput. Mol. Sci. 14, e1714. 10.1002/wcms.1714

[B30] Nagaoka M. Suzuki Y. Okamoto T. Takenaka N. (2013). A hybrid MC/MD reaction method with rare event-driving mechanism: atomistic realization of 2-chlorobutane racemization process in DMF solution. Chem. Phys. Lett. 583, 80–86. 10.1016/j.cplett.2013.08.017

[B31] Nagaoka M. Takayanagi M. Takenaka N. Suzuki Y. Matsumoto K. Koga N. (2019). “Computational molecular technology toward macroscopic chemical phenomena: Red moon methodology and its related applications,” in Molecular Technology: life innovation (Weinheim, Germany: Wiley-VCH Verlag GmbH and Co), 201–234.

[B32] Okabe T. Takehara T. Inose K. Hirano N. Nishikawa M. Uehara T. (2013). Curing reaction of epoxy resin composed of mixed base resin and curing agent: experiments and molecular simulation. Polym. (Guildf.) 54, 4660–4668. 10.1016/j.polymer.2013.06.026

[B33] Oya Y. Nakazawa M. Shirasu K. Hino Y. Inuyama K. Kikugawa G. (2021). Molecular dynamics simulation of cross-linking processes and material properties for epoxy resins using first-principle calculation combined with global reaction route mapping algorithms. Chem. Phys. Lett. 762, 138104. 10.1016/j.cplett.2020.138104

[B34] Rappé A. K. Skiff W. M. Casewit C. J. (2000). Modeling metal-catalyzed olefin polymerization. Chem. Rev. 100, 1435–1456. 10.1021/cr9902493 11749270

[B35] Resconi L. Cavallo L. Fait A. Piemontesi F. (2000). Selectivity in propene polymerization with metallocene catalysts. Chem. Rev. 100, 1253–1346. 10.1021/cr9804691 11749266

[B36] Rowley C. N. Woo T. K. (2011). Counteranion effects on the zirconocene polymerization catalyst olefin complex from QM/MM molecular dynamics simulations. Organometallics 30, 2071–2074. 10.1021/om101188t

[B37] Senftle T. P. Hong S. Islam M. M. Kylasa S. B. Zheng Y. Shin Y. K. (2016). The ReaxFF reactive force-field: development, applications and future directions. Npj Comput. Mater. 2, 15011–15014. 10.1038/npjcompumats.2015.11

[B38] Sinn H. Kaminsky W. Vollmer H.-J. Woldt R. (1980). “living polymers” on polymerization with extremely productive Ziegler catalysts. Angew. Chem. Int. Ed. Engl. 19, 390–392. 10.1002/anie.198003901

[B39] Suzuki Y. Nagaoka M. (2017). A transformation theory of stochastic evolution in Red Moon methodology to time evolution of chemical reaction process in the full atomistic system. J. Chem. Phys. 146, 204102. 10.1063/1.4983396 28571340

[B40] Takaba H. Hayashi S. Zhong H. Malani H. Suzuki A. Sahnoun R. (2008). Development of the reaction time accelerating molecular dynamics method for simulation of chemical reaction. Appl. Surf. Sci. 254, 7955–7958. 10.1016/j.apsusc.2008.04.009

[B41] Tomasi S. Razavi A. Ziegler T. (2007). Density functional theory investigation into the stereocontrol of the syndiospecific polymerization of propylene catalyzed by*C* _s_-symmetric zirconocenes. Organometallics 26, 2024–2036. 10.1021/om060786v

[B42] Unke O. T. Chmiela S. Sauceda H. E. Gastegger M. Poltavsky I. Schütt K. T. (2021). Machine learning force fields. Chem. Rev. 121, 10142–10186. 10.1021/acs.chemrev.0c01111 33705118 PMC8391964

[B43] van Duin A. C. T. Dasgupta S. Lorant F. Goddard W. A. (2001). ReaxFF: a reactive force field for hydrocarbons. J. Phys. Chem. A 105, 9396–9409. 10.1021/jp004368u

[B44] Wilke G. (2003). Fifty years of Ziegler catalysts: consequences and development of an invention. Angew. Chem. Int. Ed. Engl. 42, 5000–5008. 10.1002/anie.200330056 14595621

[B45] Yang S.-Y. Ziegler T. (2006). Combined Car−Parrinello QM/MM dynamic study on the propagation and termination steps of ethylene polymerization catalyzed by [Cp2ZrR(μ-me)B(C6F5)3] (R = me, pr). Organometallics 25, 887–900. 10.1021/om050840s

[B46] Zaccaria F. Cipullo R. Budzelaar P. H. M. Busico V. Ehm C. (2017). Backbone rearrangement during olefin capture as the rate limiting step in molecular olefin polymerization catalysis and its effect on comonomer affinity. J. Polym. Sci. A Polym. Chem. 55, 2807–2814. 10.1002/pola.28685

[B47] Ziegler T. Vanka K. Xu Z. (2005). The influence of the counterion B(C_6_F_5_)_3_CH_3_– and solvent effects on the propagation and termination steps of ethylene polymerization catalyzed by Cp_2_ZrR^+^ (R = Me,Pr). A density functional study. C. R. Chim. 8, 1552–1565. 10.1016/j.crci.2004.10.036

[B48] Zuccaccia C. Busico V. Cipullo R. Talarico G. Froese R. D. J. Vosejpka P. C. (2009). On the first insertion of α-olefins in hafnium pyridyl-amido polymerization catalysts. Organometallics 28, 5445–5458. 10.1021/om900705v

[B49] Zuccaccia C. Macchioni A. Busico V. Cipullo R. Talarico G. Alfano F. (2008). Intra- and intermolecular NMR studies on the activation of arylcyclometallated hafnium pyridyl-amido olefin polymerization precatalysts. J. Am. Chem. Soc. 130, 10354–10368. 10.1021/ja802072n 18613668

[B50] Zurek E. Ziegler T. (2003). A theoretical study of the insertion barrier of MAO (methylaluminoxane)-activated, Cp2ZrMe2-catalyzed ethylene polymerization: further evidence for the structural assignment of active and dormant species. Faraday Discuss. 124, 93–109. 10.1039/b209455j 14527212

